# Study of Carbon Matrix Composite as Wear-Resistant Plate Material on Improving Wear Resistance and Mixing Effect in Mixing Process

**DOI:** 10.3390/polym14194207

**Published:** 2022-10-07

**Authors:** Yiren Pan, Yihui Chen, Yi Pan, Junxiu Xue, Wenwen Han, Huiguang Bian

**Affiliations:** 1 College of Electromechanical Engineering, Qingdao University of Science and Technology, Qingdao 266061, China; 2 National Engineering Laboratory of Advanced Tire Equipment and Key Materials, Qingdao University of Science & Technology, Qingdao 266061, China

**Keywords:** carbon matrix composite, wear-resistant plate, friction and wear, effective friction

## Abstract

Silica and carbon black are the most important reinforcing systems in rubber formula. In the process of continuous optimization of the formula, silica gradually replaces carbon black by its characteristics. In view of the wear problem of the components of the mixer chamber caused by the increase in the proportion of silica in the formula, this research applied carbon matrix composite (CMC) materials to wear-resistant plate materials, and compared them with common wear-resistant (CWR) plate materials to explore the impact of replacing CWR plate with CMC on improving wear resistance and mixing effect. The results showed that compared with the CWR plate, CMC wear-resistant plate showed characteristics of a high friction coefficient and low wear rate (reduced by about 23%) in the mixing process of silica compound. However, the friction behavior of carbon black compound and carbon matrix composite wear-resistant plate showed an opposite trend, where the friction coefficient and wear rate increased simultaneously, especially the wear rate that increased by about 35%. The main reasons for the experimental results were related to the characteristics, elemental composition and surface morphology of carbon matrix composite, silica and carbon black. The experimental results also indicated that the carbon matrix composite wear-resistant plate is more suitable for a silica mixing process, and the increasing friction coefficient with decreasing wear rate of wear-resistant plate can further improve the importance of effective friction in mixing and prolonging the service life of wear-resistant plate.

## 1. Introduction

Carbon matrix composite (CMC) is the new engineering material with special properties, which is composed of carbon or graphite fibers as the reinforcements and carbon or graphite as the matrix [1]. CMCs are almost entirely composed of carbon, so they can withstand high temperature and a great heating rate. They have strong resistance to thermal shock and fire induction, and good chemical inertness [2]. CMCs also have excellent wear resistance and high thermal conductivity, which is often used in aerospace, aircraft, automobiles, forklift blades and bearings. In continuous research, it is found that CMCs not only have the advantages of other composites, but also have many unique features, for example: (1) The whole system is composed of carbon elements. Due to the strong affinity between carbon atoms, the CMCs have good stability at low and high temperatures; (2) The friction resistance and wear resistance are excellent; the friction coefficient is small, and the performance is stable. It is the best candidate material for various wear-resistant and friction components [3].

**Figure 1:**
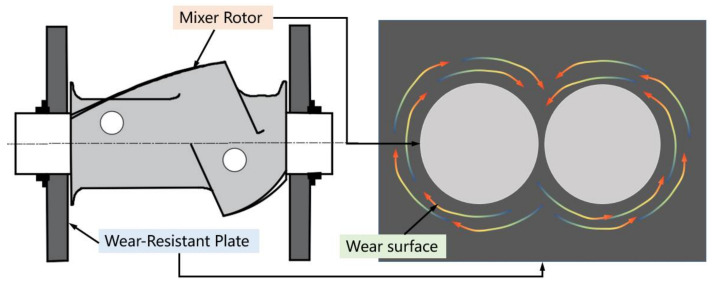
Location of wear-resistant plate and the surface wear reason.

The wear-resistant plate is an important part of the mixer. In the mixing process, it will be subjected to strong friction and squeezing of compound, and the stress condition is quite complex. The wear-resistant plate is installed at both ends of the mixing chamber, and the main function of wear-resistant plate is to locate the rotor shaft (which is shown in Figure 1); the seal and static ring can be embedded together in wear-resistant plates to improve the sealing effect of the end face and prevent the leakage of powder and rubber. In the mixing process, based on the rotor structure, the contact between compound and wear-resistant plate is mostly like the ”∞” or “○” type. When the filler and rubber are added to the mixer, the internal mixing pressure will be formed based on the filling coefficient, so that the rubber and filler will be in pressure contact with the wear-resistant plate and belt to form dry friction or mixed friction. Due to different heat-treat processes, the surface hardness of wear-resistant plate is usually less than that of internal mixing chamber walls [4]. After a long time of use, cracks appear on the surface of the wear plate. When the wear-resistant plate is worn, the surface peeling material will enter the compound, affecting the final mixing quality and the quality of rubber products [5,6].

Carbon black (CB) has been widely used as the principal reinforcement element in the rubber industry for more than a century, and a large amount of CB is added to the tire formula to improve the tire performance. Due to the small particle size and high structure of CB, when added to the compound the viscosity of the compound will be improved. Therefore, in the mixing process, the adhesive and hysteresis friction between the compound and the wear-resistant plate will lead to the wear of that plate. However, other fillers will be added or increased in the formula to enhance certain characteristics of rubber products according to the product performance requirements, which will cause friction and wear with wear-resistant plates [7,8]. When the wear reaches a certain extent, the increased clearance between the wear-resistant plate and the rotor will cause fillers uneven dispersion, ultimately affecting the comprehensive mechanical properties of the rubber.

In the 1990s, for first time, Michelin used silica instead of CB to strengthen the tread tire compound. The test results showed that compared with CB filled tire, on the premise of maintaining the original performance of the tire the rolling resistance of the silica filled tire was reduced by 20–25%, and the fuel consumption of automobiles was reduced by 6%. In addition, the safety and comfort of the car were also greatly improved. Michelin defined silica-filled tires as green tires. Although the tire filled with silica has the advantages on environmental protection, energy-saving and low rolling resistance, etc. [9,10,11], it is difficult to disperse evenly in the polymer due to the small particle size, a large amount of hydroxyl Si-OH on the surface, high polarity, easy agglomeration and poor compatibility with the compound. At the same time, the great hardness and easy agglomeration of silica will aggravate the wear of wear-resistant plate in the mixing process [12]. To improve the dispersion of silica in the rubber, a silane coupling agent was added to modify the surface of the silica in the mixing process [13,14]. In the modification process, a large amount of alcohol and water will be produced, which will cause alcohol vapor corrosion on the surface of wear-resistant plate. The addition of silica will increase the viscosity of the rubber, produce adhesion and lagging friction in the mixing process, and also cause wear on the wear-resistant plate [15,16,17]. However, the metal powder generated by the surface wear of the wear-resistant plate will enter the rubber mixing process, affecting the quality of silica rubber products. Meanwhile, the surface wear of the wear-resistant plate will lead to the powder in the mixing process becoming easier to enter the rotor end face seal, resulting in an increase in the wear of the dynamic and static ring and the leakage of the material.

This study focused on the effect of CMC used in the wear-resistant plate of the mixer on improving wear resistance and mixing effect. We set the same experimental conditions, compared friction and wear characteristics with common wear-resistant plate materials, explored the difference between the CMC and common wear-resistant plate on friction coefficient and wear resistance in silica-based or CB-based mixing, characterized the relationship between friction coefficient and wear rate, and studied the advantages of CMCs as the wear-resistant plate of mixer.

## 2. Materials and Methods

### 2.1. Materials

This section describes the rubber/filler used in the formula and the surface morphology of the two wear-resistant plate materials in the experiment.

Natural Rubber (NR): TSR20, Thailand 20# Standard Rubber, Thailand; Polymerized Styrene–Butadiene Rubber (SBR): RC2557S with 57% the content of vinyl, products of PetroChina Dushanzi Petrochemical Company, China; cis-polybutadiene (short for BR): BR9000, products of PetroChina Dushanzi Petrochemical Company, China; Carbon black (short for CB): N330, Cabot, America; Silica: Silica115MP, products of Rodia; Silane Coupling Agent: Si69mix, products of Nanjing Shuguang Chemical Group, China; 1,3-Diphenylguanidine(short for DPG): Guiechem; The protective system and activation system are all qualified products approved by the industry.

**Figure 2:**
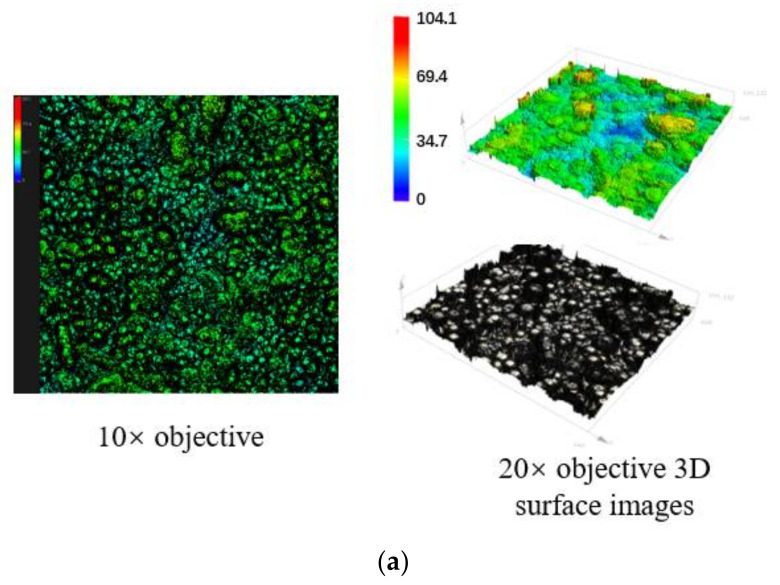

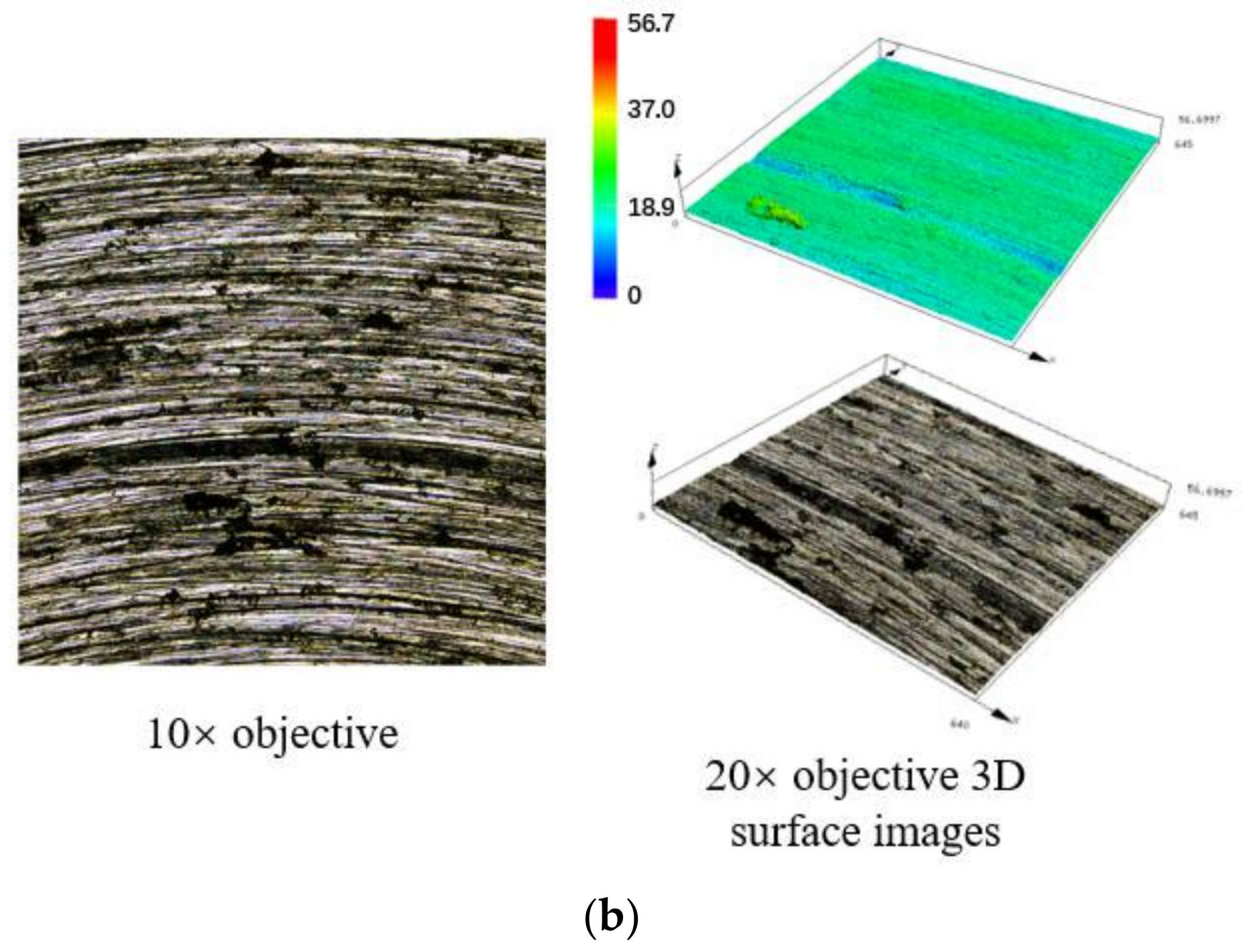
The surface of two kinds of wear-resistant plate materials: (**a**) Carbon matrix composite wear-resistant plate material; (**b**) Common wear-resistant plate material.

Two kinds of wear-resistant plate materials were used: CMC wear-resistant plate material and common wear-resistant plate material. The surface morphology of the two materials is shown in Figure 2. The surface of the carbon matrix composite wear-resistant plate material is concave–convex, with micro-gaps, micropores, and raised pillars visible. The surface of common wear-resistant plate material is flat.

### 2.2. Experiment Method

The experimental method shows the compound formula, mixing equipment, transformation method of friction test mode between compound and two kinds of wear-resistant plate materials, and the equipment and test process for the wear experiment used in this manuscript.

(1) Experimental formula and preparation

The experiment test formula is shown in Table 1.

The sample was mixed using the Hackmie Machine developed by Qingdao University of Science and Technology. Three kinds of rubber were used in the formula to better disperse the silica filler and reduce the impact on the experiment. Fill factor and rotor speed were kept constant at 0.75 and 70 rpm, respectively. Samples were taken at the middle and later stages of mixing for friction test and material surface wear analysis.

(2) Friction and wear tests

**Figure 3:**
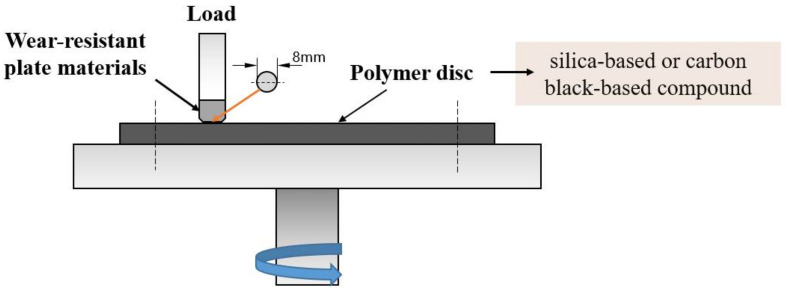
The test mode.

According to the contact mode between the rubber and internal mixing chamber wall in the mixing process and based on the principle of force interaction in Newton’s third law, simplify the contact model between rubber and metal into a pin-on-disc contact rotation experimental model, and the schematic contact mode of the frictional couple is shown in Figure 3. The upper block was two kinds of wear-resistant plate materials, while the lower disc was silica-based or CB-based compound. To ensure the accuracy of the data, the friction test was performed under the same rotation test conditions calculated by the mixing process: 150 min divided into five parts and 30 min test time each time, linear speed of 8.81 cm/s, normal loads of 8 N, test temperature of 120 °C (in the temperature effect experiment, set the test temperature as 90 °C, 110 °C, 120 °C and 140 °C). Before each test the metal surface was wiped with alcohol, and the test points were marked on the metal surface to record the coordinates of the test points, which is convenient for observation of the surface morphology after the friction test. The friction coefficient was continuously recorded by an online data acquisition system attached to the Tribometer of Anton Paar, Austria (short for CSM), and the friction coefficient was the average value during each part of the friction test.

After the friction test, the worn surfaces data and images of two kinds of disc (compound and wear-resistant plate materials) were examined using a three-dimensional morphology observation instrument (Olympus 4500, Japan) to track the sliding trajectory; magnifying the test point at the same position with a 20× objective lens observes the changes in surface morphology before and after the friction test, and the surface wear rate of the mixer chamber wall material was calculated. The calculation of surface morphology test and wear rate test is shown in Equations (1)–(4):

(1) $$\mathrm{Sa}=\frac{1}{A}\iint_{0}^{A}\left|Z\left(x,y\right)\mathrm{dxdy}\right|$$

(2) $$\mathrm{Sp}=\max z\left(x,y\right)$$

(3) $$\mathrm{Sv}=\left|\max z\left(x,y\right)\right|$$

Wear rate = V/Fnl (cm^3^/Nm) (4)

where V is the wear volume loss (mm^3^); l is the sliding distance (mm) of very circle; F is the load (N); and n is number of circles.

## 3. Results and Discussion

### 3.1. Comparison of Average Friction Coefficient

The experimental setup was found to provide repeatable results allowing for clear comparison between performances of CMC and common wear-resistant plates.

**Figure 4:**
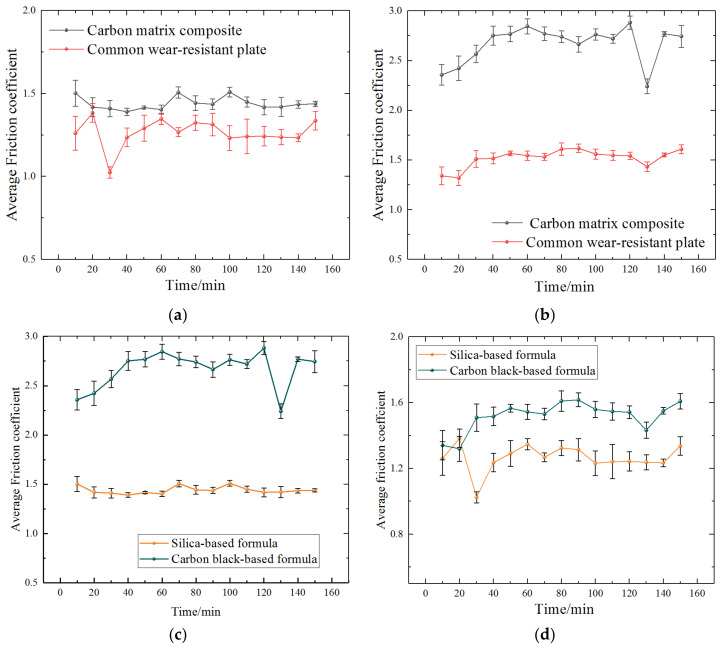
Comparison of average friction coefficient: (**a**) silica-based formula; (**b**) carbon black-based formula; (**c**) carbon matrix composite; (**d**) common wear-resistant plate.

Friction coefficient data of the different tested materials over the entire test period of 0–150 min are shown in Figure 4. By comparing the friction coefficient between silica-based compound or CB-based compound and two kinds of wear-resistant plate materials (CMC and common wear-resistant plate) in the mixing process, the results showed that the friction coefficients between silica-based compound and two kinds of wear-resistant plate materials are significantly lower than that of CB.

From the dynamic evolution, it is evident that the friction coefficient of the CMC wear-resistant plate was significantly higher than that of the common wear-resistant plate. Comparing Figure 4a,b, the curve exhibited a substantial increase in friction coefficient (~0.51) in silica-based formula and (~1.30) in CB-based formula, which is related to material properties and material interactions. Comparing Figure 4c,d, the difference in friction coefficients between CMC wear-resistant plate and the two formulas compound was significantly higher than that between the common wear-resistant plate and two formulas compound.

### 3.2. Effect of Temperature on Friction Coefficient of Two Kinds of Wear-Resistant Plate Materials and Compound

**Figure 5:**
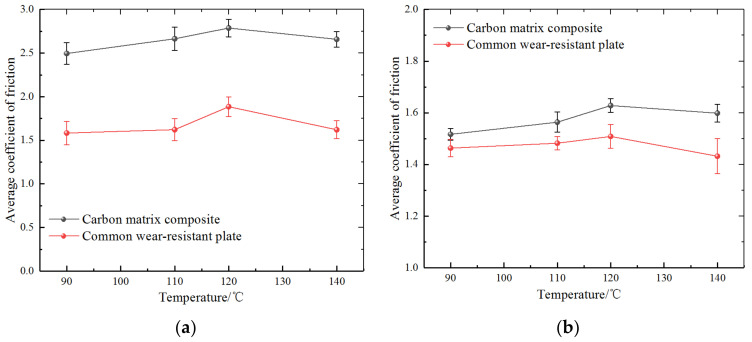
Effect of temperature on friction coefficient of two kinds of wear-resistant materials and two kinds of compound formulations: (**a**) carbon black-based formula; (**b**) silica-based formula.

Figure 5 shows the effect of temperature on the friction properties of two wear-resistant plate materials and two rubber formulas during mixing. During the mixing process the temperature rise is mainly caused by the friction between rubber and mixer chamber wall, rubber and rotor, and the internal molecular chain movement in rubber, which is helpful to the completion of the mixing process. It can be seen from the curve that with the increase in temperature, the friction coefficient of the two kinds of wear-resistant plate materials and the two formulas compound shows the same trend, that is, the trend of rising first and then falling. The reason for this trend based on the CB-based formula is that the mixing temperature is increasing during the mixing process, and the filler is gradually refined from the large particle aggregation and dispersed into the compound. The increase in mixing temperature will inevitably bring about the change in viscoelasticity of rubber. At the same time, the aggregate crushing refinement leads to an increase in viscosity of the compound. Therefore, in the CB-based formula, the friction coefficient between the two kinds of wear-resistant plate materials and the CB-based compound presented a rising tendency. As the mixing temperature continues to increase in the mixing process, the filler was uniformly dispersed in the rubber, and finally the rubber in the mixing chamber was stable, so the friction coefficient decreased. For silica-based compounds, the friction coefficient between the two kinds of wear-resistant plate materials and the compound was significantly smaller than that of the CB-based compound, which is mainly related to the characteristics and surface morphology of silica. As the temperature was raised to 140 °C, silanization reaction occurred between the silica and silane coupling agent, producing ethanol and water vapor and resulting in a decrease in friction coefficient.

Comparing the two kinds of wear-resistant plate materials, during the process of temperature change, the friction coefficient of CMC under CB-based compound mixing was significantly higher than that of the common wear-resistant plate materials. The higher friction coefficient is conducive to the strong tensile effect of the compound when passing through the wear-resistant plate. At the same time, for the later stage of mixing, the friction coefficient of the CMC decline trend was slower than for common wear-plate material, reflecting the compound mixing advantages of CMC. Especially when the silane coupling reaction occurred in the mixing of silica-based compound, the friction coefficient of CMC wear-resistant plate decreased slowly, which can better support the silica compound mixing at this stage and reduce the influence of alcohol water vapor on the mixing effect.

### 3.3. Wear Rate

The wear rate is the characterization of the surface change in the wear-resistant plate after the rubber and the wear-resistant plate friction in the mixing process. The surface wear of two kinds of wear-resistant plate materials in CB or silica formula mixing process was studied by setting the same mixing operation time of the internal mixer.

Table 2 shows the surface wear rate of CMC wear-resistant plate and common wear-resistant plate with the increase in mixing working hours. By analyzing the friction coefficient and comparing the surface wear rate of the two wear-resistant plate materials, it was found that in the mixing of silica-based formula, the wear rate of CMC and common wear-resistant plate showed the opposite trend with the friction coefficient. Compared with the wear rate of the common wear-resistant plate surface, the wear rate of the CMC surface decreased by 23.1% in the same working hours (From the data, it is found that the wear rate of the friction test experiment shows an upward trend after 120 min), and the wear rate was relatively stable during the same service period. In the mixing of CB-based formula, the wear rate of CMC and common wear-resistant plate showed the same trend as that of frictional coefficient, but the friction coefficient between CMC wear-resistant plate and CB compound was higher than that of common wear-resistant plate. Similarly, after the internal mixer run for some time, the wear rate of CMC was significantly greater than that of common wear-resistant plate, and the wear rate was about 1.5 times that of common wear-resistant plate. This was a very interesting phenomenon.

Similarly, under the same experimental conditions, comparing the wear of two kinds of wear-resistant plate materials in the mixing process of silica compound and CB compound, the results showed that when the CMC was selected as the wear-resistant plate material of mixer, the wear rate caused by silica compound mixing was less than that by CB compound mixing, which was about 11%; in the test of common wear-resistant plate, the wear rate caused by the mixing process of silica compound is greater than that of CB compound.

### 3.4. Effect of Silica-Based Compound Mixing on CMC Wear-Resistant Plate

**Figure 6:**
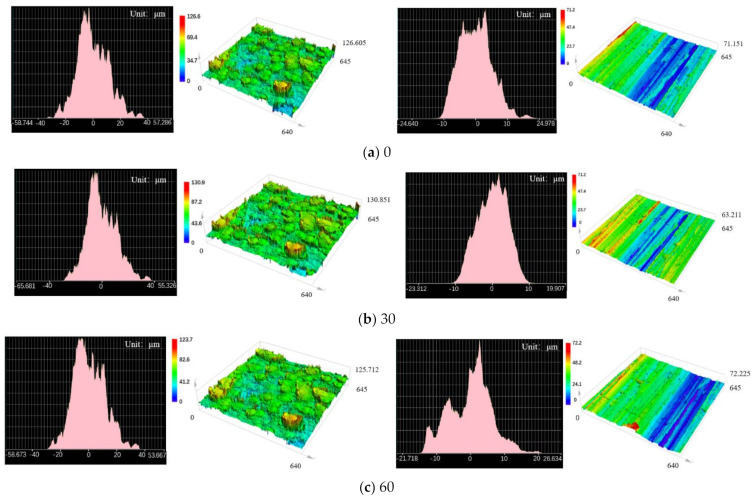

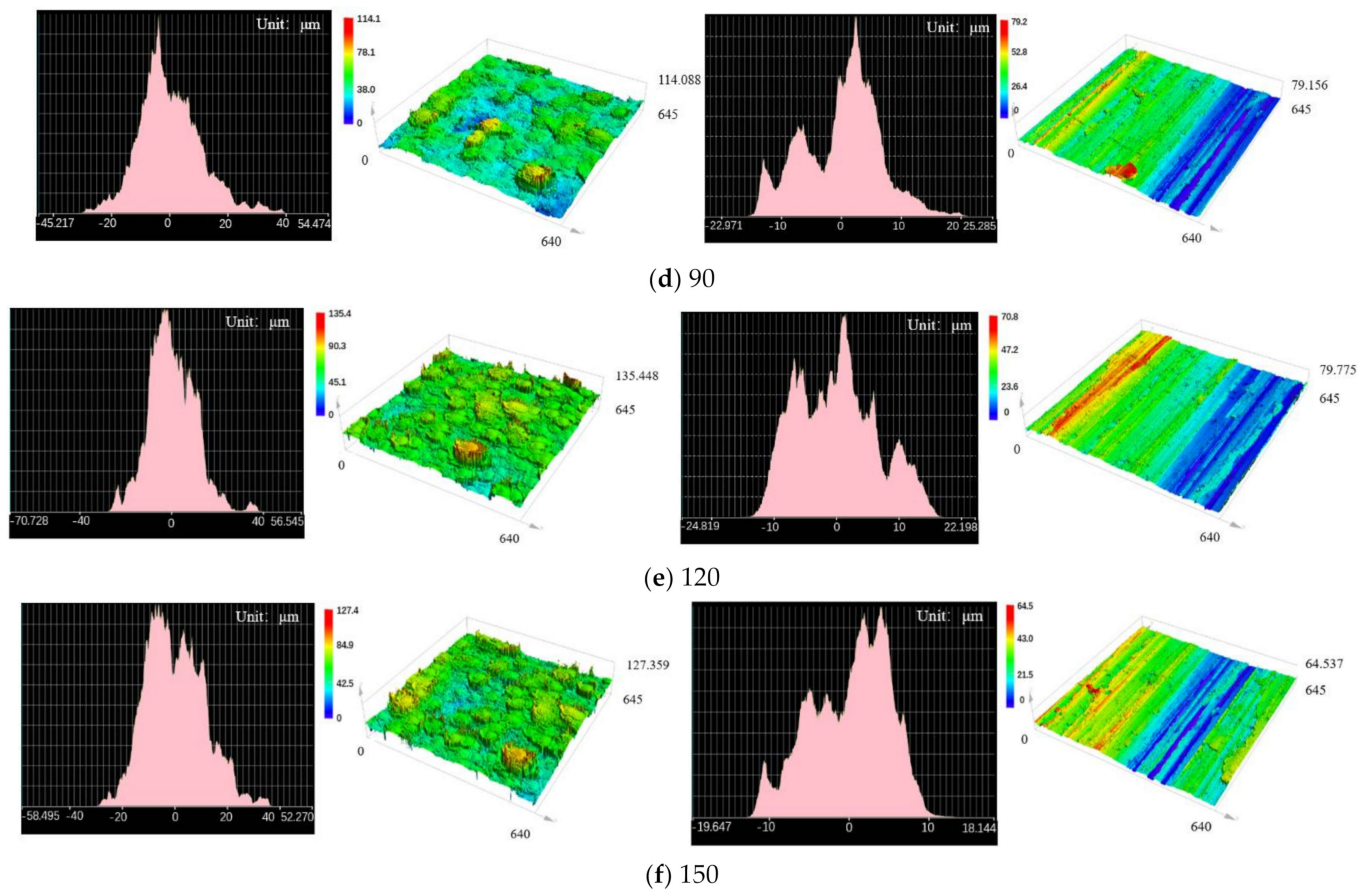
Comparison of Surface Morphology Changes (left: carbon matrix composite wear-resistant plate; right: common wear-resistant plate, Unit: μm).

**Figure 7:**
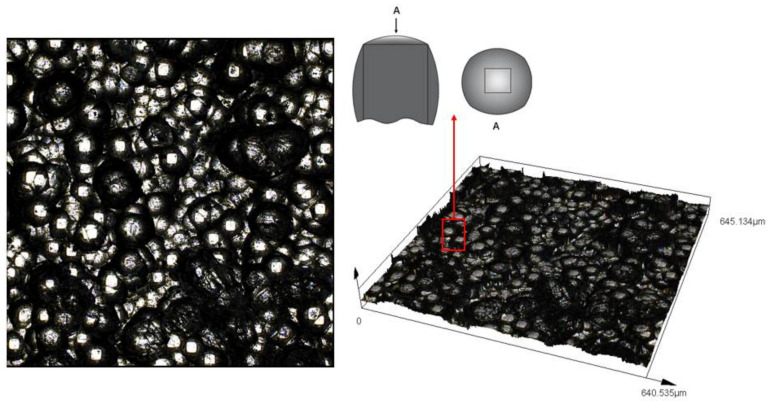
Surface Morphology of Carbon Matrix Composite.

Figure 6 shows the surface wear comparison of two wear-resistance plate materials after the same working time of silica compound mixing. With the increasing working time, after the continuous mixing of silica compound, the surface of CMC wear-resistant plate and common wear-resistant plate appeared to wear, resulting in the change in surface appearance. Combined with the two-dimensional and three-dimensional surface (as shown in Figure 6), it was found that the CMC presented a cylindrical aggregation shape after treatment and the contact surface with the rubber is shown in Figure 7A. The contact mode between CMC wear-resistant plate and silica compound was mainly high–low convex surface contact. Therefore, the wear position is mostly concentrated on the convex surface with large mixing pressure. In the mixing process, wear-resistant plates were located at the front and rear ends of the rotor (internal mixing chamber), and the contact force with the silica compound was mainly affected by the filling coefficient and axial thrust force generated by the rotor on the rubber during the mixing process. When the silica compound entered the wedge-shaped three-dimensional area formed by the rotor, the internal mixing chamber wall and the wear-resistant plate, the increasing force led to the significant wear of the wear-resistant plate. For the common wear-resistant plate, the surface was mainly plane with micro-vertical stripes, which was caused by the heat-treatment process. The contact between silica compound and common wear-resistant plate was mainly surface-to-surface contact, and the large contact area led to larger wear. It was well known that friction behavior was the most important factor in the mixing process. During the mixing process, the friction between fillers or molecular chains would produce the temperature rise, improve the viscous flow state of the compound, and promote the dispersion of fillers. The squeezing friction between mixing components and compound would cause the mutual overlap, rolling and integration of rubber, which can improve the mutual dispersion between rubber and filler and is an important factor to improve the quality of the mixing compound. Comparing the friction coefficient, wear data and surface images, the average friction coefficient between the CMC wear-resistant plate and silica compound was higher than that of the common wear-resistant plate material, while the wear rate was less than that of the common wear-resistant plate material, and the wear rate in each period test time was relatively average. In other words, the friction behavior between silica compound and the wear-resistant plate was mainly composed of adhesion friction, lag friction and particle friction in the mixing process. The larger friction coefficient reflected the stronger friction behavior between compound and the wear-resistant plate. Further, the larger contact force between compound and the wear-resistant plate caused stronger tensile deformation. This tensile deformation was affected by the filler dispersion and the chain connection between silica and rubber after the silica silane coupling, which further explained the advantages of CMC wear-resistant plate in improving the mixing effect. According to the morphology observation, the large tensile deformation was also related to the surface morphology of CMC. The images show that the surface of the CMC presents a micro columnar shape, which likes a uniformly arranged small brush in the mixing process, conducive to rubber long-chain fracture and filler dispersion. At the same time, the small amount of wear indicated that the CMC was more wear-resistant than common wear-resistant plate, which reduced the outflow of rubber or filler along the gap between the rotor and the wear-resistant plate caused by the wear-resistant plate and affected the mixing effect and the quality of rubber products.

### 3.5. Effect of Carbon Black-Based Compound Mixing on CMC Wear-Resistant Plate

**Figure 8:**
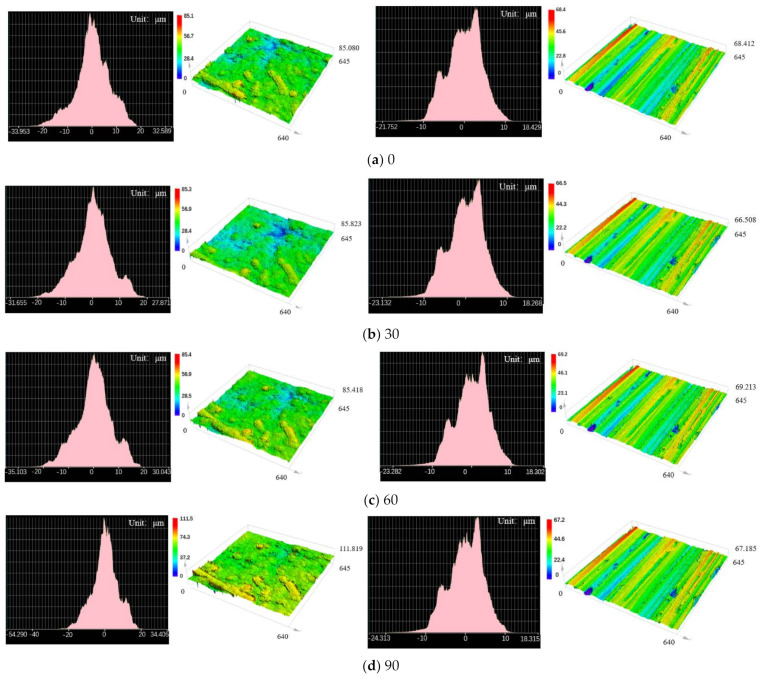

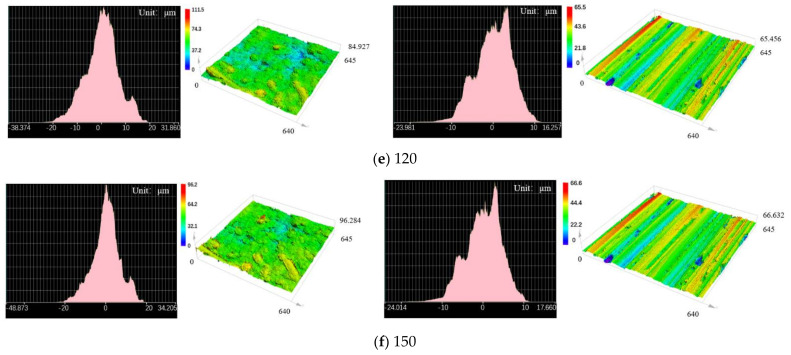
Comparison of Surface Morphology Changes (left: carbon matrix composite wear-resistant plate; right: common wear-resistant plate, Unit: μm).

**Figure 9:**
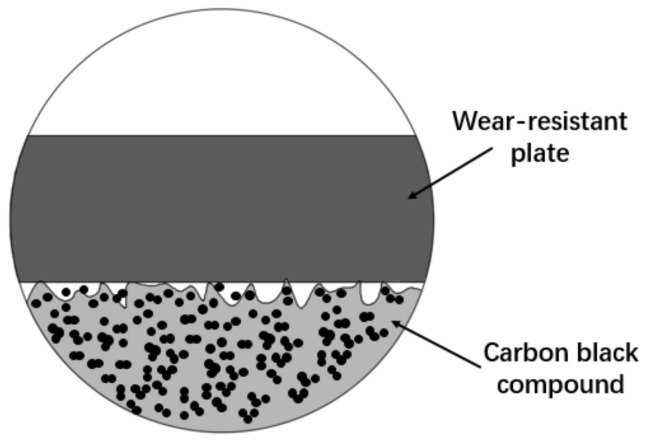
Carbon black compound and CMC attract each other.

Figure 8 shows the comparison of surface wear of two kinds of wear-resistant plates after working for the same time in CB compound mixing. In the process of CB compound mixing, combined with two-dimensional and three-dimensional surface morphology images, the surface morphology of the CMC changed more obviously than common wear-resistant plates. By comprehensively comparing the friction coefficient, wear rate and surface wear images, the friction coefficient between CB compound and CMC wear-resistant plate was higher than that of common wear-resistant plate material, about 1.5 times that of common wear-resistant plate. This comparison result was opposite to that of silica compound mixing. At the same time, the wear of common wear-resistant plate caused by the CB compound mixing process changed more evenly with the increase in equipment running time, while the wear of CMC changed to ‘wavy’ with the increase in equipment running time. Analyzing this phenomenon, we thought that the composition of the CMC was the main reason for the increase in wear rate. In the process of CB compound mixing, due to the high temperature (120–145 °C, and element attraction is related to temperature) the CB in the compound and the carbon element on the wear-resistant plate were accelerated to attract each other and the principle is shown in Figure 9. With the viscoelastic properties of rubber and the ability of mutual attraction between elements in the mixing process, the wear of wear-resistant plate was aggravated based on original friction and wear between raw rubber and wear-resistant plate.

**Figure 10:**
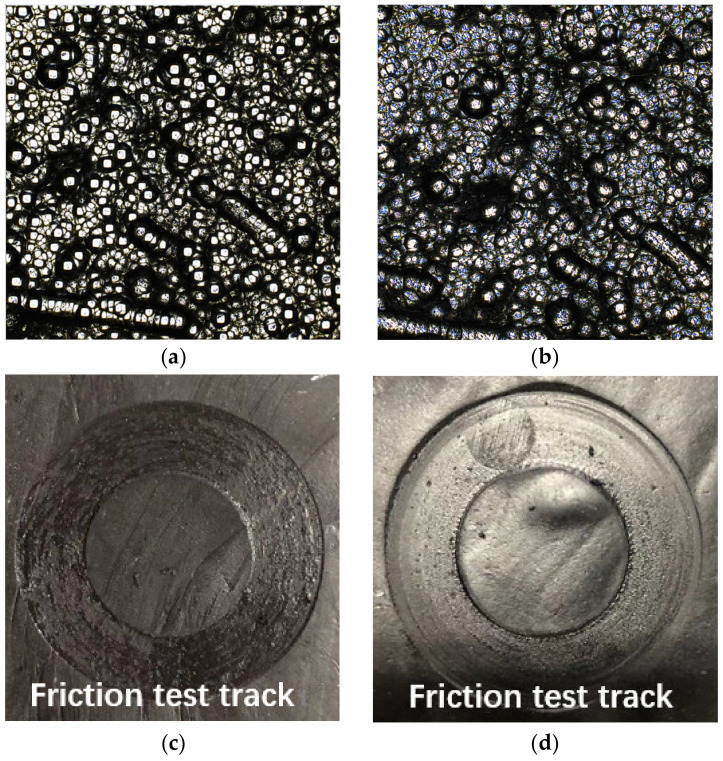
Comparison of surface morphology after friction test (above: carbon matrix composite; below: rubber sample)); (**a**) Before friction test, (**b**) After friction test, (**c**) common wear-resistant plate, (**d**) carbon matrix composite wear-resistant plate.

At the same time, it was also found that in the friction test the CMC can absorb some industrial oil on the surface of CB during the friction process. Pan Yiren’s research on friction and wear in the mixing process showed that the residual industrial oil (through the light transmittance experiment of toluene, it can be confirmed that there is an oil film on the surface of the CB particles) on the surface of CB can lubricate and protect the internal mixing chamber wall during the mixing process. Similarly, a comparable phenomenon occurred during the friction between CMC and CB compound during the mixing process in this study, where CMC absorbs the residual industrial oil on the surface of CB. Figure 10 shows the comparison of a before/after friction test of CMC wear-resistant plate, and the surface images of a CB compound sample friction track at the same test time (left: common wear-resistant plate; right: CMC wear-resistant plate). After observing the surface test of the compound after the friction experiment, the test sample surface on the right was relatively smooth, and only a few adhesion and lagging hillock bumps were generated due to the contact between the viscoelastic properties of the rubber and the rigid material; the surface oil was less, showing a dumb state. However, the test sample surface on the left had a lot of obvious adhesion convex and oil.

**Figure 11:**
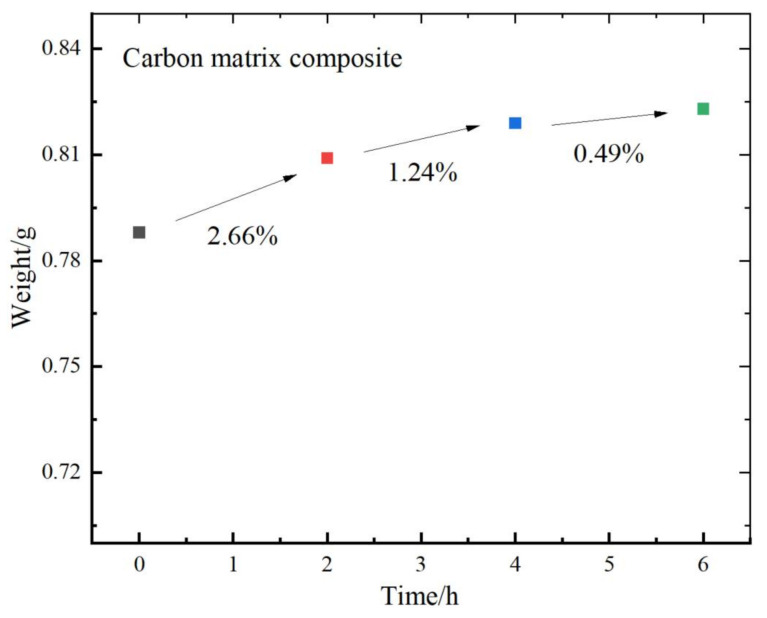
Relationship between time and weight.

Combined with the rubber surface morphology image by two kinds of wear-resistant plate materials and CB-based compound friction test, we initially believed that the CMC has oil absorption characteristics. Therefore, we also verified it by immersing two kinds of wear-resistant plate materials into the operating oil and recording the quality changes in the two kinds of wear-resistant plate materials every two hours, which is shown in Figure 11.

The surface morphology of the CMC has voids, micropores and microgaps between high and low fluctuations. Combined with the image and the quality change curve, oil molecules are mainly concentrated in the root of CMC. It can be explained that during the CB compound mixing process, the oil will also diffuse or penetrate into the CMC through micropores and micro-gaps. This phenomenon can explain that the CMC has oil absorption, and it is also a reason for accelerated wear. The left side of the sample surface friction test trajectory has a lot of obvious adhesion convex, surface oil. This phenomenon can explain that the CMC has oil absorption characteristics, and that is another reason for accelerated wear.

In conclusion, these two factors were the main reasons for the large wear rate of CMC wear-resistant plate in the process of CB mixing.

### 3.6. Comparison of Friction and Wear between Different Formulas and Two Kinds of Wear-Resistant Plate Materials

Compared with common wear-resistant plate materials, the friction coefficient between CB or silica-based formula and CMC was significantly higher. The friction coefficient between CB-based compound and the CMC was higher than that between silica-based formula and CMC, and the wear produced by CB compound on carbon matrix materials is slightly greater than that of silica compound. The results showed that the reason for this phenomenon is related to the characteristics of carbon-based materials and the two reinforcement systems.

**Figure 12:**
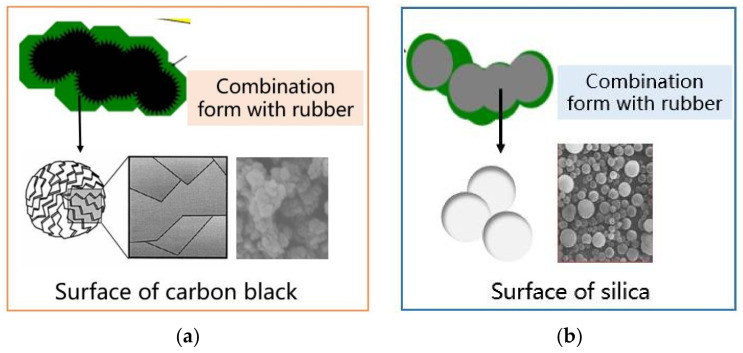
Surface morphology of carbon black and silica: (**a**) Carbon black; (**b**) Silica.

Figure 12 shows the surface morphology of CB and silica. The surface of CB presents a corrugated sawtooth shape, which still exists after being combined with rubber. Therefore, the friction behavior between CB compound and CMC wear-resistant plate included: (1) viscoelasticity compound and carbon matrix materials; (2) the corrugated serrated surface of the CB and CMC; and (3) the mutual attraction between CMC and CB increased the adhesion friction behavior. Therefore, in the mixing process, the friction coefficient of the two was large. Due to the special surface morphology of CB, its friction with the wear-resistant plate was very similar to the blade scraping friction behavior. The wear caused by the viscoelastic change in the compound and the mutual attraction between the materials were the main reasons for the high wear rate of CMC wear-resistant plate in the process of CB mixing.

In view of the friction behavior between silica-based compound and the CMC wear-resistant plate in the mixing process, due to the spherical shape and smooth surface of silica particles, it can play a similar role as a lubricant in the friction process with CMC wear-resistant plate, which was the reason why the friction coefficient was lower than that of CB compound in the whole mixing process. Different from CB mixing, when silica and rubber infiltrate each other without a silane coupling reaction, silica mainly plays the role of filler, and aggregate particles would be partially exposed outside the rubber, which is shown in Figure 12b. In addition, the hardness of silica aggregate particles is large, which is one of the reasons for the wear of carbon-based material wear-resistant plate caused by silica compound mixing. When silane coupling reaction occurred in the process of silica mixing, gaseous water and ethanol would reduce the friction coefficient of the compound and CMC wear-resistant plate. However, silane coupling reaction products would cause corrosion wear on the wear-resistant plate. Although the corrosion resistance of CMC wear-resistant plate was strong, the surface would also produce corrosion wear under the silica mixing for a long time.

## 4. Conclusions

This paper focused on studying CMC wear-resistant plate in the mixer on improving the wear resistance and mixing effect in the mixing process. Under the same test conditions, it was found that the friction coefficient between CB-based or silica-based compound and CMC wear-resistant plate was higher than that of ordinary wear-resistant plate material. The friction coefficient difference between the CB-based rubber and two kinds of wear-resistant plate materials was large, and the friction coefficient difference between the silica-based rubber and the wear-resistant plates of the two materials was small. Comparing the total wear rate of two kinds of wear-resistant plate in the same equipment running time, the wear rate of CMC wear-resistant plate was less than that of common wear-resistant plate material in the silica-based formula mixing, and the wear loss is reduced by about 23%. While the opposite result appeared in the CB-based mixing, the wear rate of CMC wear-resistant plate material was higher than that of common wear-resistant plate, and the wear loss was increased by about 35%. The results also showed that the friction coefficients and wear rates of CB-based and wear-resistant plate were higher than those of silica-based mixing. According to the experimental results, it is obvious that the CMC wear-resistant plate was more suitable for silica-based mixing equipment. The increasing friction coefficient and decreasing wear rate can further improve the effective friction mixing effect. The main reasons for this experimental result can be analyzed as follows:

(1) Characteristics of CMC, CB and silica. The different surface morphology of CB particles and silica particles caused the difference in contact with CMC wear-resistant plate. The former increased friction and accelerated wear, and the latter reduced friction and reduced wear. At the same time, the oil absorption of the CMC and the mutual attraction between the materials accelerated the wear during CB mixing;
(2) Surface morphology of CMC. In the mixing process of the silica compound, compared with the surface morphology of common wear-resistant plate, the surface of the CMC presented the shape of cylindrical aggregation with a gap. Furthermore, this morphology played a tensile mixing effect, promoted the rolling flow of the compound, and reduced its contact wear;
(3) Corrosion resistance of CMC. In the mixing process of the silica compound, the compound performance can be enhanced by silane coupling reaction, which will produce the mixed vapor of water and alcohol with corrosion damage. The corrosion resistance of CMC can reduce the abrasion of alcohol vapor caused by silane coupling reaction to improve the service life of wear-resistant plate.

## Figures and Tables

**Table 1:** Sample Formula (Unit: phr).

Raw Material	Carbon Black-Based	Silica-Based
BR9000	25.5	25.5
RC2557S	81.81	81.81
TSR20	15	15
N330	70	10
Silica115MP	/	60
Si69mix	/	7.2
DPG	1	0.8
Others	Protection system: 3.5 phr; Activation system: 4 phr	

**Table 2:** Wear rate of two kinds of materials(10^−12^·cm^3^·Nm^−1^).

Time/Min	Silica-Based Compound		CB-Based Compound	
	CMC	Common Wear-Resistant Plate	CMC	Common Wear-Resistant Plate
0				
30	531.545	314.156	168.096	309.077
60	537.028	556.053	350.999	332.259
90	310.643	356.495	955.537	324.800
120	555.769	754.185	472.947	340.573
150	631.711	1356.517	895.778	490.263
Sum	2566.696	3337.406	2843.358	1796.972

## Data Availability

Not applicable.
